# Skew Convolutional Codes

**DOI:** 10.3390/e22121364

**Published:** 2020-12-02

**Authors:** Vladimir Sidorenko, Wenhui Li, Onur Günlü, Gerhard Kramer

**Affiliations:** 1 Institute for Communications Engineering, Technical University of Munich, 80333 München, Germany; gerhard.kramer@tum.de; 2 Skolkovo Institute of Science and Technology, 143026 Moscow, Russia; w.li@skoltech.ru; 3 Information Theory and Applications Chair, Technical University of Berlin, 10623 Berlin, Germany; guenlue@tu-berlin.de

**Keywords:** convolutional codes, skew polynomials, time-varying codes, dual codes, trellises

## Abstract

A new class of convolutional codes, called skew convolutional codes, that extends the class of classical fixed convolutional codes, is proposed. Skew convolutional codes can be represented as periodic time-varying convolutional codes but have a description as compact as fixed convolutional codes. Designs of generator and parity check matrices, encoders, and code trellises for skew convolutional codes and their duals are shown. For memoryless channels, one can apply Viterbi or BCJR decoding algorithms, or a dualized BCJR algorithm, to decode skew convolutional codes.

## 1. Introduction

Convolutional codes were introduced by Elias in 1955 [1]. With the discovery that convolutional codes can be decoded with Fano sequential decoding [2], Massey threshold decoding [3], and, above all, Viterbi decoding [4], they became quite widespread in practice. Convolutional codes are still widely used in telecommunications, e.g., in Turbo codes [5] and in the WiFi IEEE 802.11 standard [6], in cryptography [7], etc.

The most common are binary convolutional codes; however, communication with higher orders of modulation [8] or streaming of data [9] require *non-binary convolutional codes*. It is known that *periodic time-varying convolutional codes improve the free distance and weight distribution over fixed convolutional codes*; see, e.g., Mooser [10] and Lee [11]. This is the motivation to introduce a new class of periodic time-varying non-binary convolutional codes, i.e., skew convolutional codes. These codes are based on the non-commutative ring of skew polynomials over finite fields and on the skew field of their fractions.

Block codes based on skew polynomials were studied by various authors; see, e.g., publications of Gabidulin [12], Boucher and Ulmer [13,14], Martínez-Peñas [15], Gluesing-Luerssen [16], Abualrub, Ghrayeb, Aydin, and Siap [17].

Convolutional codes are nonblock linear codes over a finite field, but it can be advantageous to treat them as block codes over certain infinite fields. We will use both approaches. Classical convolutional codes are described by usual polynomials. The product of the polynomials corresponds to convolution of vectors of their coefficients and this gives fixed-in-time classical convolutional codes. We replace usual polynomials by skew polynomials to define the new codes. The product of skew polynomials corresponds to skew convolution of their coefficients, which can be obtained by varying elements in the usual convolution. In this way, we obtain time varying convolutional codes.

Our goal is to define and to give a first encounter with skew convolutional codes. In Section 2, we define skew convolutional codes. In Section 3, we obtain generator matrices and encoders for skew codes and show that skew codes are equivalent to time-varying convolutional codes. Some useful properties of skew codes are considered in Section 4. Section 5 introduces dual skew convolutional codes. Trellis decoding of skew codes is considered in Section 6. Section 7 concludes the paper.

## 2. Skew Convolutional Codes

### 2.1. Skew Polynomials and Fractions

Consider a field F and an automorphism θ of the field. Later on, we will use the finite field $F=F_{q^{m}}$, where *q* is a prime power, with the *Frobenius automorphism*

(1) $$\theta \left(a\right)=a^{q},\;\forall a\in F.$$

The composition of automorphisms is denoted as $\theta \left(\theta \left(a\right)\right)=\theta^{2}\left(a\right)$, and, for any integer *i*, we have $\theta^{i}\left(a\right)=\theta \left(\theta^{i-1}\left(a\right)\right)$. The identity automorphism θ(a)=a is denoted as θ=id. For the automorphism (1) for all a∈F, we have $\theta^{i}\left(a\right)=a^{q^{i}}$ and $\theta^{m}=\mathrm{id}$ since $a^{q^{m}}=a$.

Denote by R=F[D;θ] the non- commutative ring [18] of *skew polynomials* in the variable *D* over F (with zero derivation) such that

$$R=F\left[D;\theta \right]=\{a\left(D\right)=a_{0}+a_{1}D+\cdots +a_{n}D^{n}\;|\;a_{i}\in F\mathrm{and}n\in N\}.$$

The skew polynomials look like usual polynomials F[D] where the coefficients $a_{i}$ are placed to the right of the variable *D*. The addition in R is as for usual polynomials from F[D]. The multiplication is defined by the basic rule

(2) Da=θ(a)D

and is extended to all elements of R by associativity and distributivity; see Example 1 below. The ring R has a unique left skew field of fractions Q, from which it inherits its linear algebra properties, see, e.g., [19] for more details.

**Example** **1.** *To demonstrate our results, we use the field $F_{Q}=F_{q^{m}}=F_{2^{2}}$, q=2, m=2, with automorphism $\theta \left(a\right)=a^{q}=a^{2}$ for all $a\in F_{2^{2}}$. The field $F_{2^{2}}$ consists of the elements $\left\{0,1,\alpha ,\alpha^{2}\right\}$, where a primitive element α satisfies $\alpha^{2}+\alpha +1=0$ and the following relations hold:*
$\alpha^{2}=\alpha +1\;\alpha^{3}=1,\;\alpha^{4}=\alpha$,
$and\;\forall i\in Z\;\theta^{i}=\left\{\begin{array}{cc} \theta & if\;i\;is\;odd,\\ id & if\;i\;is\;even. \end{array}\right.$,

*Let a(D)=1+αD and $b\left(D\right)=\alpha^{2}+D$, a(D),b(D)∈R. Using (2), we compute the product ab as*

$$\;a\left(D\right)b\left(D\right)=\left(1+\alpha D\right)\left(\alpha^{2}+D\right)=\left(\alpha^{2}+D\right)+\alpha \theta \left(\alpha^{2}\right)D+\alpha D^{2}=\alpha^{2}+\alpha D+\alpha D^{2}$$

*while the product ba is*

$$b\left(D\right)a\left(D\right)=\left(\alpha^{2}+D\right)\left(1+\alpha D\right)=\left(\alpha^{2}+D\right)+\alpha^{3}D+\theta \left(\alpha \right)D^{2}=\alpha^{2}+\alpha^{2}D^{2}.$$

*In this example, we see that a(D)b(D)≠b(D)a(D).*

*The left skew field Q consists of fractions $\frac{b(D)}{a(D)}=a^{-1}\left(D\right)b\left(D\right)\in Q$ for all a(D),b(D)∈R, a(D)≠0. Every fraction can be expanded as the left skew Laurent series in increasing powers of D. In our example, the inverse element $\frac{1}{a(D)}=a{\left(D\right)}^{-1}$ is expanded using long devision as follows:*

$$a^{-1}\left(D\right)=1+\alpha D+D^{2}+\alpha D^{3}+D^{4}+\ldots$$

*with $a^{-1}\left(D\right)a\left(D\right)=a\left(D\right)a^{-1}\left(D\right)=1$. We can expand the left fraction $\frac{b(D)}{a(D)}=a^{-1}\left(D\right)b\left(D\right)$ by long left division or equivalently by left multiplication b(D) by $a^{-1}\left(D\right)$ and get*

$$\frac{b(D)}{a(D)}=a^{-1}\left(D\right)b\left(D\right)=\alpha^{2}+\alpha D+D^{2}+\alpha D^{3}+D^{4}+\ldots \;.$$

*Notice that the right fraction $b\left(D\right)a^{-1}\left(D\right)=\alpha^{2}$, since $b\left(D\right)=\alpha^{2}+D=\alpha^{2}\left(1+\alpha D\right)=\alpha^{2}a\left(D\right)$.*

### 2.2. Definition of Skew Convolutional Codes

Much of linear algebra can be generalized from vector spaces over a field to (either left or right) modules over the skew field Q. Indeed, it is shown in [19] that any left Q-module C is free, i.e., it has a basis, and any two bases of C have the same cardinality, the dimension of C.

**Definition** **1** (Skew convolutional code)**.** *Given an automorphism θ of the field F, a skew convolutional [n,k] code C over the field F is a left sub-module of dimension k of the free module $Q^{n}$.*

The elements of the code C are called its *codewords*. A codeword $v\left(D\right)=\left(v^{\left(1\right)}\left(D\right),\ldots ,v^{\left(n\right)}\left(D\right)\right)$ is an *n*-tuple over Q, where every component $v^{\left(i\right)}\left(D\right)$ is a fraction of skew polynomials from R. The code C is $F=F_{q^{m}}$-linear. Let the weight of every codeword be defined by some selected metric. The *free distance* $d_{f}$ of a skew convolutional code is defined to be the minimum nonzero weight of any codeword.

For the Hamming metric, which is the most interesting for applications, the weight of a fraction $v^{\left(i\right)}\left(D\right)$ is the number of nonzero coefficients in its expansion as a left skew Laurent series from F((D)) in increasing powers of *D*. The weight of a codeword is the sum of weights of its components. Another interesting metric is sum-rank metric, which will be defined later.

### 2.3. Relations to Fixed Convolutional Codes

**Lemma** **1.** *The class of skew convolutional codes includes the class of fixed (time-invariant) convolutional codes.*

**Proof.**  A time-invariant convolutional [n,k] code $\tilde{C}$ over the field F is defined as a *k*-dimensional subspace of $F{\left(D\right)}^{n}$. Take the identity automorphism θ=id. Then, the ring R=F[D;θ] becomes a ring F[D] of usual commutative polynomials. The skew field of fractions Q becomes the field of rational functions F(D). In this case, by Definition 1, the skew convolutional code C coincides with the classical fixed code $\tilde{C}$. □

## 3. Encoding Skew Convolutional Codes

### 3.1. Polynomial Form of Encoding

A *generator matrix* of a skew convolutional [n,k] code C is a k×n matrix G(D) over the skew field Q whose rows form a basis for the code C. If the matrix G(D) is over the ring R of skew polynomials, then G(D) is called a *polynomial generator matrix* for C. Every skew code C has a polynomial generator matrix. Indeed, given a generator matrix G(D) over the skew field of fractions Q, a polynomial generator matrix can be obtained by left multiplying each row of G(D) by the least common left multiple of the denominators in that row. In the paper, we focus on polynomial generator matrices and corresponding encoders.

By Definition 1, every codeword v(D) of a skew code C, which is an *n*-tuple over the skew field of fractions Q

(3) $$v\left(D\right)=\left(v^{\left(1\right)}\left(D\right),\;v^{\left(2\right)}\left(D\right),\ldots ,\;v^{\left(n\right)}\left(D\right)\right),\;v^{\left(j\right)}\left(D\right)\in Q,\;1\leq j\leq n$$

can be written as

(4) v(D)=u(D)G(D)

where u(D) is a *k*-tuple (*k*-word) over Q:

(5) $$u\left(D\right)=\left(u^{\left(1\right)}\left(D\right),\;u^{\left(2\right)}\left(D\right)\ldots ,\;u^{\left(k\right)}\left(D\right)\right),\;u^{\left(i\right)}\left(D\right)\in Q,\;1\leq i\leq k$$

and is called an information word, G(D) is a generator matrix of C, $G\left(D\right)\in Q^{k\times n}$ or $G\left(D\right)\in R^{k\times n}$. Relation (4) already provides an encoder. This encoder (4) is just an encoder of a block code over Q and the skew code C can be considered as the set of *n*-tuples v(D) over Q that satisfy (4), i.e., C={v(D)}.

However, to use the code in practice we need to write components of u(D) and v(D) as skew Laurent series, i.e., we have

(6) $$u^{\left(i\right)}\left(D\right)=u_{0}^{\left(i\right)}+u_{1}^{\left(i\right)}D+u_{2}^{\left(i\right)}D^{2}+\ldots ,\;i=1,\ldots ,k$$

and

(7) $$v^{\left(j\right)}\left(D\right)=v_{0}^{\left(j\right)}+v_{1}^{\left(j\right)}D+u_{2}^{\left(j\right)}D^{2}+\ldots ,\;j=1,\ldots ,n.$$

Actually, in a Laurent series, the lower (time) index of coefficients can be a negative integer, but, in practice, the information sequence $u^{\left(i\right)}\left(D\right)$ should be causal for every component *i*, that is, the coefficients $u_{t}^{\left(i\right)}$ are zeros for time t<0. Causal information sequences should be encoded into causal code sequences; otherwise, an encoder can not be implemented, since it should output code symbols before it receives an information symbol.

Denote the block of information symbols that enters an encoder at time t=0,1,… by

(8) $$u_{t}=\left(u_{t}^{\left(1\right)},u_{t}^{\left(2\right)},\ldots u_{t}^{\left(k\right)}\right)\in F^{k}.$$

The block of code symbols that leaves the encoder at time t=0,1,… is denoted by

(9) $$v_{t}=\left(v_{t}^{\left(1\right)},v_{t}^{\left(2\right)},\ldots v_{t}^{\left(n\right)}\right)\in F^{n}.$$

Combining (5), (6), and (8), we obtain the following information series with vector coefficients:

(10) $$u\left(D\right)=u_{0}+u_{1}D+\ldots +u_{t}D^{t}+\ldots ,\;u\left(D\right)\in F{\left(\left(D\right)\right)}^{k}.$$

Using (3), (7), and (9), we write a codeword as a series

(11) $$v\left(D\right)=v_{0}+v_{1}D+\ldots +v_{t}D^{t}+\ldots ,\;v\left(D\right)\in F{\left(\left(D\right)\right)}^{n}.$$

One can write a skew polynomial generator matrix $G\left(D\right)=\left(g_{ij}\left(D\right)\right)\in R^{k\times n}$ as a skew polynomial with matrix coefficients:

(12) $$G\left(D\right)=G_{0}+G_{1}D+G_{2}D^{2}+\ldots +G_{\mu }D^{\mu }$$

where μ is the maximum degree of polynomials $g_{ij}\left(D\right)$. The matrices $G_{i}$ are k×n matrices over the field F and μ is called the generator matrix *memory*.

**Figure 1:**
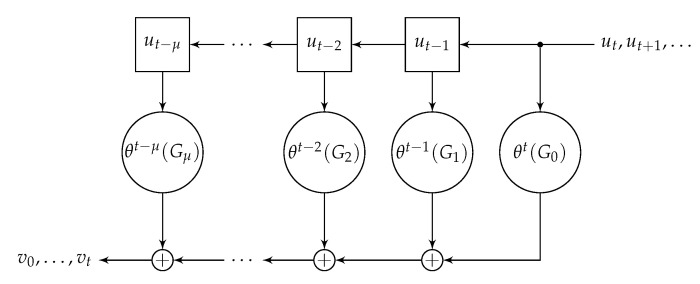
Encoder of a skew convolutional code.

From (4), (10), and (11), we obtain that $v_{t}$ is a coefficient in the product of skew series u(D) and skew polynomial G(D), which is the following *skew convolution* (see Figure 1)

(13) $$v_{t}=u_{t}\theta^{t}\left(G_{0}\right)+u_{t-1}\theta^{t-1}\left(G_{1}\right)+\cdots +u_{t-\mu }\theta^{t-\mu }\left(G_{\mu }\right)$$

where $u_{t}=0$ for t<0. This encoding rule explains the title *skew convolutional code*, which can be also seen as the set C={v(D)} of series v(D) defined in (11).

At time *t*, the decoder observes an information block $u_{t}$ of *k* symbols from F and outputs the code block $v_{t}$ of *n* code symbols from F using (13); hence, the *code rate* is R=k/n. The encoder (13) uses $u_{t}$ and also μ previous information blocks $u_{t-1},u_{t-2},\ldots ,u_{t-\mu }$, which should be stored in the encoder’s memory; this is why μ is also the encoder *memory*.

The coefficients $\theta^{t-i}\left(G_{i}\right)$, i=0,1,…,μ in the encoder (13) depend on the time *t*. Hence, the *skew convolutional code is a time-varying classical convolutional code*. Denote

(14) $$\tau =\min\left\{i>0\;:\;\theta^{i}\left(G_{j}\right)=G_{j}\;\;\forall j=0,1,\ldots ,\mu \right\}.$$

For the field $F=F_{q^{m}}$, we have $\theta^{m}=\theta^{0}$; hence, the coefficients in (13) are periodic with period τ≤m, and the *skew convolutional code is periodic with period τ≤m*. The period τ can be less than *m* if all the matrices $G_{0},\ldots G_{\mu }$ are over a subfield of $F_{q^{m}}$.

### 3.2. Scalar Form of Encoding

The input of the encoder can also be written as an information sequence of *k*-blocks over F

(15) $$u=u_{0},\;u_{1},\;u_{2},\ldots ,u_{t},\ldots \;$$

and the output as a code sequence of *n*-blocks over F

(16) $$v=v_{0},\;v_{1},\;v_{2},\ldots ,\;v_{t},\;\ldots \;.$$

Then, the encoding rule (13) can be written in a scalar form

(17) v=uG

with semi-infinite scalar generator block matrix

(18) $$G=\left(\begin{array}{ccccccc} G_{0} & G_{1} & G_{2} & \cdots & G_{\mu } & \; & \\ \; & \theta (G_{0}) & \theta (G_{1}) & \ldots & \theta (G_{\mu -1}) & \theta (G_{\mu }) & \\ \; & & \theta^{2}\left(G_{0}\right) & \ldots & \theta^{2}\left(G_{\mu -2}\right) & \theta^{2}\left(G_{\mu -1}\right) & \theta^{2}\left(G_{\mu }\right)\\ \; & \; & & \ldots \end{array}\right).$$

Thus, a skew convolutional code can be equivalently represented in a scalar form as the set C={v} of sequences *v* defined in (16) that satisfy (17).

### 3.3. Relations between Skew and Classical Convolutional Codes

In case of identity automorphism, θ(a)=a, the scalar generator matrix of the skew code becomes

(19) $$G^{\prime }=\left(\begin{array}{ccccccc} G_{0} & G_{1} & G_{2} & \ldots & G_{\mu } & \; & \\ \; & G_{0} & G_{1} & \ldots & G_{\mu -1} & G_{\mu } & \\ \; & & G_{0} & \ldots & G_{\mu -2} & G_{\mu -1} & G_{\mu }\\ \; & \; & & \ldots \end{array}\right),$$

which is a generator matrix of a fixed convolutional code [20]. For fixed convolutional codes, polynomial generator matrices with $G_{0}$ of full rank *k* are of particular interest [20] (Chapter 3). The skew convolutional codes use the following nice property: if $G_{0}$ has full rank, then $\theta^{i}\left(G_{0}\right)$ has full rank as well for all *i*.

Time-varying classical convolutional codes are defined by the following generator matrices in [20],

(20) $$G_{var}=\left(\begin{array}{cccccc} G_{0,0} & G_{1,1} & \; & G_{\mu ,\mu } & \; & \\ \; & G_{1,0} & \vdots & G_{\mu ,\mu -1} & G_{\mu +1,\mu }\\ \; & & \; & \vdots & G_{\mu +1,\mu -1} & \vdots \\ \; & \; & & G_{\mu ,0} & \vdots \\ \; & \; & & \; & G_{\mu +1,0} \end{array}\right)$$

where the first index *t* in $G_{t,i}$ is time index. The code defined by the generator matrix $G_{var}$ in (20) is called *τ-periodic* if the columns ${\left(G_{t,\mu }^{T},\ldots ,G_{t,0}^{T}\right)}^{T}$, t≥μ, repeat with period τ.

**Lemma** **2.** *A scalar generator matrix (18) of a skew code can be written in the following equivalent form:*

(21) $$G=\left(\begin{array}{cccccc} {\tilde{G}}_{0} & \theta ({\tilde{G}}_{1}) & \; & \theta^{\mu }\left({\tilde{G}}_{\mu }\right) & \; & \\ \; & \theta ({\tilde{G}}_{0}) & \vdots & \theta^{\mu }\left({\tilde{G}}_{\mu -1}\right) & \theta^{\mu +1}\left({\tilde{G}}_{\mu }\right) & \\ \; & & \; & \vdots & \theta^{\mu +1}\left({\tilde{G}}_{\mu -1}\right) & \vdots \\ \; & \; & & \theta^{\mu }\left({\tilde{G}}_{0}\right) & \vdots \\ \; & \; & & \; & \theta^{\mu +1}\left({\tilde{G}}_{0}\right) \end{array}\right).$$

**Proof.**  The statement follows from the change of variables $G_{i}=\theta^{i}\left({\tilde{G}}_{i}\right)$ for i=1,2,…,μ. □

From (14), (20) and (21), we see again that a skew code defined by a generator matrix (21) is a τ-periodic classical convolutional code. Thus, above we proved the following theorem.

**Theorem** **1.** *Given a field $F=F_{q^{m}}$ with automorphism θ in (1), any skew convolutional [n,k] code C over F is equivalent to a periodic time-varying (classical) convolutional [n,k] code over F, with period τ≤m (14). If G(D) is a skew polynomial generator matrix (12) of the code C, then the scalar generator matrix G of the time-varying code is given by (18) or (21).*

Not every periodic classical convolutional code can be represented as a skew code. Indeed, e.g., the submatrix $G_{1,0}$ in (20) can be selected independently of $G_{0,0}$ while corresponding submatrix $\theta ({\tilde{G}}_{0})$ in (21) is completely determined by ${\tilde{G}}_{0}$. Hence, a class of skew convolutional codes is a subclass of periodic classical convolutional codes.

Given the field $F_{q^{m}}$, the automorphism θ in (1), and the code memory μ, an [n,k] skew convolutional code is defined by a generator matrix *G* in (21). To specify the matrix, we should fix elements of μ+1 matrices ${\tilde{G}}_{0},\ldots ,{\tilde{G}}_{\mu }$ of size k×n. Hence, we should define (μ+1)kn field elements. Since a classical convolutional code corresponds to the identity automorphism θ=id, the *description of skew and classical codes require the same number of field elements*.

The number of skew [n,k] convolutional codes over $F_{Q}=F_{q^{m}}$ of memory μ with fixed automorphism $\theta \left(a\right)=a^{q}$ has order $q^{(\mu +1)mkn}$. The number of τ-periodic classical convolutional codes has order $q^{(\mu +1)mkn\tau }$, which is much larger. As a result, the search of good periodic time-varying convolutional codes is much simpler in the class of skew codes in comparison with periodic classical codes. The search among skew convolutional codes has the same complexity as the search among fixed classical codes.

How many more skew codes can we obtain by considering all possible automorphisms? Denote $q=p^{s}$, where *p* is the field characteristic then $F_{q^{m}}=F_{p^{sm}}=F_{p^{M}}$, i.e., our field $F_{Q}$ is an *M*-extension of the prime field $F_{p}$. The parameter $q=p^{s}$, we should select such that $F_{q}$ is a subfield of $F_{p^{M}}$, hence *s* should divide *M*. Denote by δ(M) the *number-of-divisors function* that is the number of divisors *i* of *M*, 1≤i≤M. Given the field $F_{Q}=F_{p^{M}}$, we can select $q=p^{i}$ and the automorphism $\theta \left(a\right)=a^{q}$ in δ(M) ways.

**Lemma** **3.** *For a fixed field $F_{Q}=F_{p^{M}}$, there are δ(M) sub-classes of skew convolutional codes, each defined by a fixed automorphism θ.*

In Example 2, we have $F_{Q}=F_{2^{2}}$, i.e., p=q=2,s=1,m=2. M=sm=2 with divisors 1 and 2. For i=1, we have $q=p^{i}=2$ and $\theta \left(a\right)=a^{2}$ considered in Example 2. For i=2, we have $q=p^{2}=4$ that corresponds to θ=id and gives a constant classical convolutional code. For the field $F_{2}^{6}$, we have δ(6)=4, and there are four sub-classes of skew codes with $q\;=\;2^{1},\;2^{2},\;2^{3}$, and $q=2^{6}$ (for fixed code).

## 4. Properties of Skew Convolutional Codes

### 4.1. Extension of Fixed Convolutional Codes

To show properties of skew convolutional codes, we will use the following example.

**Example** **2.** *Consider a [2,1] skew convolutional code $\hat{C}$ over the field $F_{Q}=F_{q^{m}}=F_{2^{2}}$ with automorphism $\theta \left(a\right)=a^{q}=a^{2}$ (see Example 1). Let the generator matrix of the code $\hat{C}$ in polynomial form be*

(22) $$G\left(D\right)=\left(1+\alpha D,\;\alpha +\alpha^{2}D\right)=G_{0}+G_{1}D,$$

*where*

(23) $$G_{0}=\left(1,\alpha \right),\;G_{1}=\left(\alpha ,\alpha^{2}\right).$$

*The generator matrix in scalar form (18) is*

(24) $$G=\left(\begin{array}{ccccc} 1\;\alpha & \alpha \;\alpha^{2}\\ \; & 1\;\alpha^{2} & \alpha^{2}\;\alpha \\ \; & \; & 1\;\alpha & \alpha \;\alpha^{2}\\ \; & \; & & 1\;\alpha^{2} & \alpha^{2}\;\alpha \\ \; & \; & \ldots \end{array}\right).$$

*Here, μ=1; hence, it is a unit memory code. The encoding rule is v=uG, or, from (13), it is*

(25) $$v_{t}=u_{t}\theta^{t}\left(G_{0}\right)+u_{t-1}\theta^{t-1}\left(G_{1}\right),\;t=0,1,\ldots \;.$$

*In some applications, it is preferable to have a generator matrix in systematic form. For our example, a systematic fractional matrix can be obtained using the left division of its components by the first component*

(26) $$G_{syst}\left(D\right)=\left(1,\frac{\alpha +\alpha^{2}D}{1+\alpha D}\right)=\left(1,{\left(1+\alpha D\right)}^{-1}\left(\alpha +\alpha^{2}D\right)\right).$$

Let us show that the matrices $G_{syst}\left(D\right)$ and G(D) in (22) encode the same code $\hat{C}$. Denote $g_{1}\left(D\right)=1+\alpha D$. Then, for any information sequence u(D)∈Q, we have the code word

$$u\left(D\right)G\left(D\right)=u\left(D\right)g_{1}\left(D\right)g_{1}^{-1}\left(D\right)G\left(D\right)=u\left(D\right)g_{1}\left(D\right)G_{syst}\left(D\right)=u^{\prime }\left(D\right)G_{syst}\left(D\right)$$

and the statement follows since there is a one to one mapping between u(D) and $u^{\prime }\left(D\right)=u\left(D\right)g_{1}\left(D\right)$; hence, both information sequences u(D) and $u^{\prime }\left(D\right)$ run over all possible causal sequences.

**Theorem** **2.** *The class of skew convolutional codes extends the class of fixed convolutional codes.*

**Proof.**  By Lemma 1, the class of fixed codes is included in the class of skew convolutional codes. Hence, it is sufficient to show that there exists a codeword in a skew convolutional [n,k] code that can not belong to any fixed [n,k] code with the same memory. Indeed, consider the unit memory, μ=1, skew [2,1] code $\hat{C}$ defined by the generator matrix (24). By encoding the information sequence u=1,0,0,1, we obtain the codeword $v=v_{0},\;v_{1},\;v_{2},\;v_{3},\;v_{4}=\left(1,\alpha \right),\left(\alpha ,\alpha^{2}\right),\left(0,0\right),\left(1,\alpha^{2}\right),\left(\alpha^{2},\alpha \right)\in \hat{C}$.

Suppose for the sake of contradiction that the codeword *v* belongs to a fixed unit memory [2,1] convolutional code $C^{\prime }$. A general form of generator matrix of a unit memory fixed [2,1] code $C^{\prime }$ is (19):

$$G^{\prime }=\left(\begin{array}{ccccc} a\;b & c\;d\\ \; & a\;b & c\;d\\ \; & \; & a\;b & c\;d\\ \; & \; & & a\;b & c\;d\\ \; & \; & \ldots \end{array}\right)$$

where $a,b,c,d,\in F_{2^{2}}$. Assume that the word $v=\left(e,f,g,h,\ldots \right)G^{\prime }\in C^{\prime }$ where $e,f,g,h\in F_{2^{2}}$. From $v_{2}=f\left(c,d\right)+g\left(a,b\right)=\left(0,0\right)$, it follows that either i) f=g=0, or ii) vectors (c,d) and (a,b) are $F_{2^{2}}$-linearly dependent. In case i), $v_{0}=e\left(a,b\right)=\left(1,\alpha \right)$ and $v_{3}=h\left(a,b\right)=\left(1,\alpha^{2}\right)$, $e^{-1}\left(1,\alpha \right)=h^{-1}\left(1,\alpha^{2}\right)$, which is impossible since the vectors (1,α) and $\left(1,\alpha^{2}\right)$ are linearly independent. In case ii), linear combinations of linearly dependent vectors (c,d) and (a,b) should give two linearly independent vectors $v_{0}=\left(1,\alpha \right)$ and $v_{3}=\left(1,\alpha^{2}\right)$, which is impossible as well. □

### 4.2. Canonical Encoders and Generator Matrices

**Figure 2:**
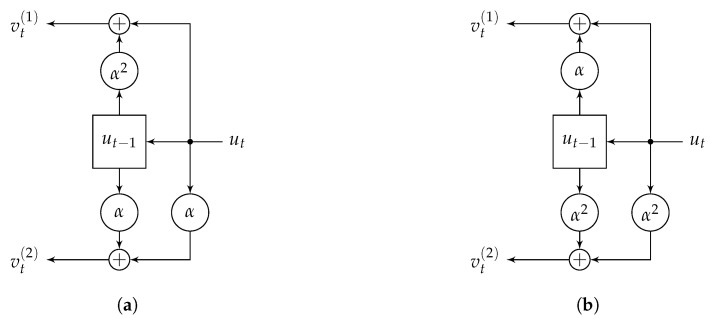
Encoder of the skew code $\hat{C}$ from Example 2: (**a**) for even *t*, (**b**) for odd *t*.

The encoder in a controller canonical form [20] for the code $\hat{C}$ in Example 2 with generator matrix (22) is shown in Figure 2a for even *t* and in Figure 2b for odd *t*. The encoder has one shift register, since k=1. There is one *Q*-ary memory element in the shift register shown as a rectangle, where $Q=q^{m}=4$ is the order of the field. We need only one memory element since a maximum degree of items in G(D), which consists of a single row in our example, is 1. A large circle means multiplication by the coefficient shown inside.

*In the general case* of a k×n matrix G(D), we define the degree $\nu_{i}$ of its *i*th row as the maximum degree of its components, and *external degree* ν of G(D) is the sum of its row degrees [21]. The controller-canonical-form encoder of G(D) over $F_{Q}$ has *k* shift registers, the *i*th register has $\nu_{i}$ memory elements, and the total number of *Q*-ary memory elements in the encoder is ν. Different generator matrices for a code C may have different external degrees.

**Definition** **2.** *Among all skew polynomial generator matrices (PGM) for a given skew convolutional code C, those for which the external degree is as small as possible are called canonical PGMs. This minimal external degree is called the degree or overall constraint length of the code C, and denoted as ν=degC.*

### 4.3. Code Trellises

**Figure 3:**
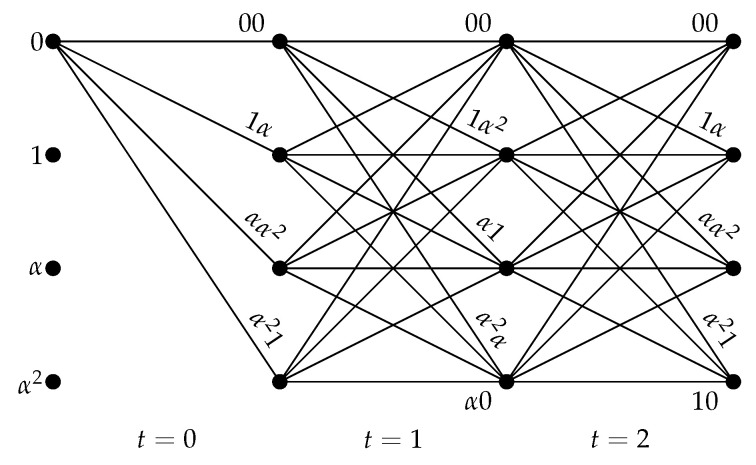
Time-varying trellis of the skew code $\hat{C}$.

For the code $\hat{C}$ in Example 2, the code trellis is shown in Figure 3. The trellis consists of sections periodically repeated with period τ=m=2. Every section has $Q^{\nu }=4^{1}=4$ states labeled by elements of the field $F_{Q}$. For the *t*-th section for time t,t=0,1,…, an edge connects the states $u_{t-1}$ and $u_{t}$ and is labeled by the code block $v_{t}$. Every codeword is represented by a path in the trellis that starts from the zero state 0 and goes to the right. The edge label $v_{t}$ is computed according to the encoding rule (25) as follows:

(27) $$v_{t}=\left\{\begin{array}{cc} u_{t-1}\left(\alpha ,\alpha^{2}\right)+u_{t}\left(1,\alpha^{2}\right) & \mathrm{for}\;\mathrm{odd}\;t,\\ u_{t-1}\left(\alpha^{2},\alpha \right)+u_{t}\left(1,\alpha \right) & \mathrm{for}\;\mathrm{even}\;t \end{array}\right.$$

where we assume that $u_{-1}=0$, i.e., the initial state of the shift register is 0.

### 4.4. Code Distances

There are two important characteristics of a convolutional code: the free distance $d_{f}$ and the slope σ of the increase of the active burst distance, defined as follows [20]. The weight of a branch labeled by a vector $v_{t}$ is defined to be the weight $w(v_{t})$ of $v_{t}$. The weight of a path is the sum of its branch weights. A path in the trellis that diverges from zero state, which does not use edges of weight 0 from zero state to zero state, and that returns to zero state after *ℓ* edges is called a loop of length *ℓ* or *ℓ-loop*.

The *ℓth order active burst distance* $d_{\ell }^{\mathrm{burst}}$ is defined [20] to be the minimum weight of *ℓ*-loops in the code trellis. The slope is defined as $\sigma =\lim_{\ell \to \infty }d_{\ell }^{\mathrm{burst}}/\ell$. The free distance is $d_{f}=\min_{\ell }d_{\ell }^{\mathrm{burst}}$.

**Theorem** **3** (Singleton bound)**.** *The free Hamming distance of [n,k] skew convolutional code C of degree ν=degC is upper bounded as follows:*

(28) $$d_{f}\leq \left(n-k\right)\left\lfloor \frac{\nu }{k}+1\right\rfloor +\nu +1.$$

**Proof.**  We adopted the proof given in [22] for time-invariant finite state codes. The trellis of the code C is time-varying with $Q^{\nu }$ states at every level. Consider $Q^{\ell k}$ information sequences $u_{0},\ldots ,u_{\ell -1}$ of length *ℓ* blocks. For each of them, the code path in the trellis starts at the state 0 and terminates in one of $Q^{\nu }$ states. From the pigeon-hole principle, it follows that there must be at least $Q^{\ell k-\nu }=Q^{K}$ of these paths that have the same final state. The code sequences corresponding to these paths can be thought of as a block code with length N=ℓn with at least $Q^{K}$ codewords. We should select *ℓ* such that K=ℓk−ν>0. The Hamming distance *d* of the block code is upper bounded by the Singleton bound d≤N−K+1=ℓ(n−k)+ν+1. On the other hand, *d* is an upper bound on the free Hamming distance $d_{f}$ of the code C. Since this is true for all ℓ>ν/k, we have

$$d_{f}\leq \min_{\ell :\;\ell >\nu /k}\ell \left(n-k\right)+\nu +1$$

that gives the upper bound (28). □

To obtain (28), we use the Singleton bound for block codes; therefore, the bound (28) is *Singleton-type bound for skew convolutional codes*. In fact, this bound and the proof are valid for arbitrary (also for nonlinear) time-varying trellis codes. In [23], codes that reach the Singleton-type bound are called maximum distance separable (MDS) codes. Any other upper bound for the Hamming distance of block codes can be used to obtain another upper bound for $d_{f}$ of skew convolutional codes (also for time-varying trellis codes). Using the Plotkin bound for block codes, we obtain the following bound.

**Corollary** **1** (Heller bound)**.** *The free Hamming distance of [n,k] skew convolutional code C over $F_{Q}$ of degree ν=degC and memory μ is upper bounded as follows:*

(29) $$d_{f}\leq \min_{i\in \hat{N}}\left\lfloor \frac{n\left(\mu +i\right)Q^{k(\mu +i)-\nu -1}\left(Q-1\right)}{Q^{k(\mu +i)-\nu }-1}\right\rfloor ,$$

*where $\hat{N}=\left\{1,2,\ldots \right\}$ if kμ=ν and $\hat{N}=\left\{0,1,\ldots \right\}$, otherwise.*

The bound is named Heller since it was obtained for fixed binary convolutional codes in 1968, see [20,24]. The bound (29) is valid for for time-varying (nonlinear or linear) trellis codes.

In the Hamming metric, the upper bound

(30) σ≤n−k

for the slope σ was obtained in [25] for fixed binary convolutional codes. We conjecture that this bound is true also for non-binary time-varying convolutional codes, and, hence, for skew convolutional codes.

Another interesting metric for convolutional codes is the *sum-rank metric*, which can be applied for multi-shot network coding [26]. The metric is defined as follows. The *rank weight* $w_{R}\left(v_{t}\right)$ of a vector $v_{t}$ over the extension field $F_{q^{m}}$ is the rank of the vector over the base field $F_{q}$, i.e., $w_{R}\left(v_{t}\right)$ is the maximum number of $F_{q}$-linearly independent components of $v_{t}$. The sum-rank weight of a sequence *v* in (16) is the sum of weights of its items $v_{t}$. The sum-rank distance between two sequences is the weight of their difference.

The rank of a vector $v_{t}\in F_{q^{m}}^{n}$ is upper bounded by the Hamming weight $w_{H}\left(v_{t}\right)$ of the vector, i.e.,

(31) $$w_{R}\left(v_{t}\right)\leq w_{H}\left(v_{t}\right).$$

Hence, any upper bound for the Hamming metric is an upper bound for the sum-rank metric, and, from Theorem 3, we have the following corollary.

**Corollary** **2.** *The free sum-rank distance $d_{f}$ of [n,k] skew convolutional code C of degree ν=degC is upper bounded by (28) or by (29).*

On the other hand, from (31), we obtain the following lemma.

**Lemma** **4.** *Let the distance of a code C in the sum-rank metric be d; then, in the Hamming metric, the code distance is at least d.*

We next compute the free distance, active burst distances, and the slope for the code $\hat{C}$ from Example 2 for the Hamming and for the sum-rank metrics.

**Lemma** **5.** *In the sum-rank metric, the skew convolutional code $\hat{C}$ defined by G(D) in (22) has the ℓ-th active burst distance $d_{\ell }^{burst}=\ell +2$ for ℓ=2,3,…, the slope of the active distance is σ=1, and the free distance is $d_{f}=4$.*

**Proof.**  For this code, the shortest length of a loop is ℓ=2; hence, we should consider loops of length ℓ=2,3,…. It follows from (27) that the weight $w_{R}\left(v_{t}\right)=\mathrm{rank}\;v_{t}$ of a branch in the code trellis that diverges from or merges to zero state is 2. A branch connecting two nonzero states has weight at least 1. Indeed, for odd *t*, the branch label $v_{t}$ in (27) is a linear combination of vectors $\left(\alpha ,\alpha^{2}\right)$ and $\left(1,\alpha^{2}\right)$ that are $F_{q^{m}}$-linearly independent, and $v_{t}$ can’t be 0 for nonzero coefficients $u_{t-1},u_{t}$. The same is true for even *t*. Hence, $d_{\ell }^{\mathrm{burst}}\geq \ell +2$. On the other hand, the path, corresponding to the information sequence $u_{0}=u_{1}=\ldots =u_{\ell -1}\neq 0$, $u_{\ell }=0$, is an *ℓ*-loop of weight ℓ+2. Hence, $d_{\ell }^{\mathrm{burst}}\leq \ell +2$, and the statement of the lemma follows. □

Combining Lemmas 3 and 4, we obtain the following corollary.

**Corollary** **3.** *In the Hamming metric, the skew convolutional code $\hat{C}$ defined by G(D) in (22) has the ℓ-th active burst distance $d_{\ell }^{burst}=\ell +2$ for ℓ=2,3,…, the slope of the active distance is σ=1 and free distance is $d_{f}=4$.*

For both metrics, the Hamming and the sum-rank, the upper bounds for the free distance (28) and for the slope (30) for the unit memory [2,1] code $\hat{C}$ become

$$d_{f}\leq 2n-k+1=4,\mathrm{and}\sigma \leq n-k=1.$$

Hence, the skew code $\hat{C}$ defined by (22) achieves the Singleton-type upper bound on $d_{f}$ and can be called MDS codes like in [23]. The Heller bound (29) gives $d_{f}\leq 4$ as well. The slope of $\hat{C}$ also reaches the upper bound (30).

A generator matrix G(D) of a skew convolutional code (and corresponding encoder) is called *catastrophic* if there exists an information sequence u(D) of infinite weight such that the code sequence v(D)=u(D)G(D) has finite weight. The generator matrix G(D) in (22) of skew convolutional code $\hat{C}$ with $\theta ={\left(.\right)}^{q}$ is non-catastrophic, since the slope σ>0. Note that, in case of fixed convolutional code $C^{\prime }$, i.e., for θ=id, the generator matrix (22) is catastrophic, and the code has $d_{f}=2$ and σ=0.

### 4.5. Blocking of Skew Convolutional Codes

A skew convolutional code C, represented as a τ-periodic [n,k] code, can be considered as a [τn,τk] *fixed* code $C^{\left(\tau \right)}$ by *τ-blocking*, described in [21]. The only difference between C and $C^{\left(\tau \right)}$ is that the code symbols are grouped in blocks $v_{t}$ of different lengths in these codes. In this way, known methods to analyze fixed codes can be applied to skew convolutional codes.

For example, the [2,1] skew code $\hat{C}$ with generator matrix (24) has period τ=m=2 and can be written as [4,2] *fixed* code $C^{\left(\tau \right)}=C^{\left(2\right)}$ defined by the scalar generator matrix

$$G^{\left(2\right)}=\left(\begin{array}{ccc} 1\;\alpha \;\alpha \;\alpha^{2} & 0\;0\;0\;0\;\\ 0\;0\;1\;\alpha & \;\alpha^{2}\;\alpha \;0\;0\\ \; & 1\;\alpha \;\alpha \;\alpha^{2} & 0\;0\;0\;0\;\\ \; & 0\;0\;1\;\alpha & \;\alpha^{2}\;\alpha \;0\;0\\ \; & \ldots \end{array}\right)$$

which coincides with the matrix *G* in (24) but is written in 2-blocked form. From $G^{\left(2\right)}$, we obtain the generator polynomial matrix of the [4,2] blocked code ${\hat{C}}^{\left(2\right)}$ as

$$G^{\left(2\right)}\left(D\right)=\left(\begin{array}{cccc} 1 & \alpha & \alpha & \alpha^{2}\\ \alpha^{2}D & \alpha D & 1 & \alpha \end{array}\right).$$

In general, for any skew convolutional code C and for any *i*-blocking $C^{\left(i\right)}$, $i\in N_{1}$, the codewords, represented by sequences *v* of elements from $F_{q^{m}}$ in (16), are the same for codes C and $C^{\left(i\right)}$. Hence, the codes have the same properties, e.g., we have

(32) $$\deg C=\deg C^{\left(i\right)}.$$

## 5. Dual Skew Convolutional Codes

### 5.1. Definitions of Duality

The duality of skew convolutional codes can be defined in different ways.

*First*, consider a skew convolutional code C over F in a scalar form as a set of sequences as in (16). For two sequences *v* and $v^{\prime }$, where at least one of them is finite, define the scalar product $\left(v,v^{\prime }\right)$ as the sum of products of corresponding components, where missing components are assumed to be zeros. We say that the sequences are orthogonal if $(v,v^{\prime })=0$.

**Definition** **3.** *The dual code $C^{⊥}$ to a skew convolutional [n,k] code C is an [n,n−k] skew convolutional code $C^{⊥}$ such that $(v,v^{⊥})=0$ for all finite length words v∈C and for all words $v^{⊥}\in C^{⊥}$.*

*Another way* to define orthogonality is, for example, as follows. Consider two *n*-words v(D) and $v^{⊥}\left(D\right)$ over $Q^{n}$. We say that $v^{⊥}\left(D\right)$ is left-orthogonal to v(D) if $v^{⊥}\left(D\right)v\left(D\right)=0$ and right-orthogonal if $v\left(D\right)v^{⊥}\left(D\right)=0$. A left dual code to a skew convolutional code C can be defined as

$$C_{\mathrm{left}}^{⊥}=\left\{v^{⊥}\in Q^{n}:v^{⊥}\left(D\right)v\left(D\right)=0\;\mathrm{for}\;\mathrm{all}\;v\in C\right\}.$$

The dual code $C_{\mathrm{left}}^{⊥}$ is a left submodule of $Q^{n}$, hence it is a skew convolutional code.

Later on, we consider dual codes according to Definition 3 only, since it is more interesting for practical applications.

### 5.2. Parity Check Matrices

Given a code C with generator matrix *G*, we next show how to find a parity check matrix *H*, such that $GH^{T}=0$.

Let a skew [n,k] code C of memory μ be defined by a polynomial generator matrix G(D) in (12), which corresponds to the scalar generator matrix *G* in (18). For the dual [n,n−k] code $C^{⊥}$, we write a transposed parity check matrix $H^{T}$ of memory $\mu^{⊥}$, similar to classical convolutional codes, as

(33) $$H^{T}=\left(\begin{array}{cccccc} H_{0}^{T} & H_{1}^{T} & \ldots & H_{\mu^{⊥}}^{T} & \; & \\ \; & \theta (H_{0}^{T}) & \ldots & \theta (H_{\mu^{⊥}-1}^{T}) & \theta (H_{\mu^{⊥}}^{T}) & \\ \; & \; & \ldots \end{array}\right)$$

where $\mathrm{rank}(H_{0})=n-k$. Similar to [20], we call the matrix $H^{⊥}$ the *syndrome former* and write it in polynomial form as

(34) $$H^{T}\left(D\right)=H_{0}^{T}+H_{1}^{T}D+\cdots +H_{\mu^{⊥}}^{T}D^{\mu^{⊥}}.$$

Then, we have the following *parity check matrix* of the causal code C with the generator matrix (21)

(35) $$H=\left(\begin{array}{ccc} H_{0}\\ H_{1} & \theta (H_{0})\\ \vdots & \vdots \\ H_{\mu^{⊥}} & \theta (H_{\mu^{⊥}-1}) & \vdots \\ \; & \theta (H_{\mu^{⊥}}) \end{array}\right)$$

which, in the case of θ= id, coincides with the check matrix of a classical fixed convolutional code.

From Definition 3, we have that $vH^{T}=0$ for all sequences v∈C over F. On the other hand, from (4), we have that every codeword v(D)∈C can be written as v(D)=u(D)G(D). Hence, if we find an n×(n−k) matrix $H^{T}\left(D\right)$ over R of full rank such that $G\left(D\right)H^{T}\left(D\right)=0$, then every codeword satisfies $v\left(D\right)H^{T}\left(D\right)=u\left(D\right)G\left(D\right)H^{T}\left(D\right)=0$ and, vice versa, i.e., if $v\left(D\right)H^{T}\left(D\right)=0$ then v(D) is a codeword of C.

**Theorem** **4.** *We have $G\left(D\right)H^{T}\left(D\right)=0$ if and only if $GH^{T}=0$.*

**Proof.**  We show the proof for the code of memory μ=1 (like in Example 2). For the general memory case, the proof follows similarly. Consider the code with generator matrices G(D) and *G* given by (12) and (18). Let us find a check matrix with memory $\mu^{⊥}=\mu =1$. Then, we have

(36) $$H^{T}\left(D\right)=H_{0}^{T}+H_{1}^{T}D$$

and

(37) $$H^{T}=\left(\begin{array}{cccc} H_{0}^{T} & H_{1}^{T}\\ \; & \theta (H_{0}^{T}) & \theta (H_{1}^{T})\\ \; & \; & \theta^{2}\left(H_{0}^{T}\right) & \theta^{2}\left(H_{1}^{T}\right)\\ \; & \; & \ldots \end{array}\right).$$

From the condition $G\left(D\right)H^{T}\left(D\right)=0$, we have the following system of equations for unknowns $H_{0}^{T},H_{1}^{T}$

(38) $$\left\{\begin{array}{c} G_{0}H_{0}^{T}=0\\ G_{0}H_{1}^{T}+G_{1}\theta \left(H_{0}^{T}\right)=0\\ G_{1}\theta \left(H_{1}^{T}\right)=0. \end{array}\right.$$

From the condition $GH^{T}=0$, we obtain the following equations: by multiplying the first row of *G* by $H^{T}$ we get the system (38), by multiplying the second row of of *G* by $H^{T}$ we get the system

$$\left\{\begin{array}{c} \theta \left(G_{0}\right)\theta \left(H_{0}^{T}\right)=0\\ \theta \left(G_{0}\right)\theta \left(H_{1}^{T}\right)+\theta \left(G_{1}\right)\theta^{2}\left(H_{0}^{T}\right)=0\\ \theta \left(G_{1}\right)\theta^{2}\left(H_{1}^{T}\right)=0 \end{array}\right.$$

which is equivalent to (38). Multiplication of other rows of *G* by $H^{T}$ does not give new equations. Hence, conditions $G\left(D\right)H^{T}\left(D\right)=0$ and $GH^{T}=0$ give the same system (38). □

**Example** **3.** *For the code $\hat{C}$ from Example 2, we write $H_{0}=\left(a,c\right)$ and $H_{1}=\left(b,d\right)$. Using $G_{0},G_{1}$ from (22) and solving the system (38), we obtain $H_{0}=\left(\alpha ,1\right)$ and $H_{1}=\left(1,\alpha \right)$. Hence, H(D)=(α+D,1+αD) and a parity check matrix H of the code $\hat{C}$, which is a generator matrix for the dual code ${\hat{C}}^{⊥}$, is as follows:*

$$H=\left(\begin{array}{cccc} \alpha \;1\\ 1\;\alpha & \alpha^{2}\;1\\ \; & 1\;\alpha^{2} & \alpha \;1 & \vdots \\ \; & \; & 1\;\alpha \end{array}\right).$$

### 5.3. Trellises of Dual Codes

Similar to fixed convolutional codes, we have the following theorem:

**Theorem** **5.** *For a skew convolutional code C and its dual $C^{⊥}$, we have $\deg C=degC^{⊥}$.*

**Proof.**  Denote by τ and $\tau^{⊥}$ periods of the codes C and $C^{⊥}$, respectively. Let *ℓ* be the least common multiple of periods τ and $\tau^{⊥}$; then, the *ℓ*-blocked codes $C^{\left(\ell \right)}$ and ${\left(C^{⊥}\right)}^{\left(\ell \right)}$ are both fixed convolutional codes. The fixed codes $C^{\left(\ell \right)}$ and ${\left(C^{⊥}\right)}^{\left(\ell \right)}$ are dual to each other, since blocking does not change code sequences, hence $\deg C^{\left(\ell \right)}=\deg{\left(C^{⊥}\right)}^{\left(\ell \right)}$, see, e.g., Theorem 2.69, [20] for fixed dual convolutional codes. From (32), we have $\deg C=C^{\left(\ell \right)}$, $\deg C^{⊥}=\deg{\left(C^{⊥}\right)}^{\left(\ell \right)}$, hence $\deg C=\deg C^{⊥}$. □

It follows from Theorem 5 that the number of states at one level of the code trellis (trellis complexity) is the same for an original code C and for its dual $C^{⊥}$ and equals $Q^{\deg C}$.

**Figure 4:**
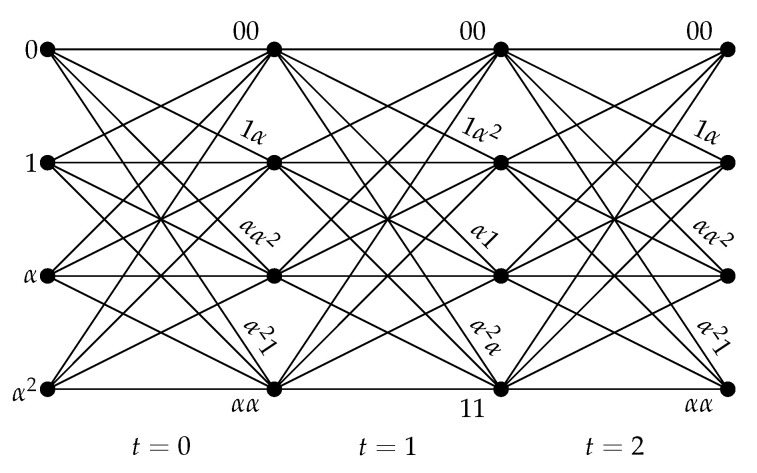
Time-varying trellis of the dual skew code ${\hat{C}}^{⊥}$ from Example 3.

The trellis of the dual code ${\hat{C}}^{⊥}$ obtained from the matrix *H* in Example 3 is shown in Figure 4. The trellis has $Q^{\deg\hat{C}}=4^{1}=4$ states labeled by elements of the set $S=\{0,1,\alpha ,\alpha^{2}\}$. Every word of the dual code ${\hat{C}}^{⊥}$ is represented by a path in the trellis that starts from a state $s_{-1}\in S$ and goes to the right. For the trellis section corresponding to time t=0,1,…, the edge connecting states $s_{t-1}$ and $s_{t}$ are labeled by $v_{t}^{⊥}$ computed as follows:

$$v_{t}^{⊥}=\left\{\begin{array}{cc} s_{t-1}\left(\alpha^{2},1\right)+s_{t}\left(1,\alpha^{2}\right) & \mathrm{for}\;\mathrm{odd}t,\\ s_{t-1}\left(\alpha ,1\right)+s_{t}\left(1,\alpha \right) & \mathrm{for}\;\mathrm{even}t. \end{array}\right.$$

## 6. Trellis Decoding of Skew Convolutional Codes

For a given skew convolutional code C, we showed how to obtain a code trellis using a generator matrix of the code. Another way to obtain a code trellis of C using a parity check matrix *H* was proposed in [27]. Having a code trellis, one can use the Viterbi decoder [4] for maximum likelihood sequence decoding or the BCJR decoder [28] for symbol-wise decoding.

For an [n,k] skew convolutional code, the complexity of the Viterbi decoder has order $\varkappa =nQ^{k}Q^{\deg C}$ operations (additions and binary selections), which exponentially increases in *k* and might be high for high rate codes. Using detailed code trellis [27,29], where every edge is labeled by a single field element, the decoding complexity can be reduced to

(39) $$\varkappa =nQ^{\min\{k,n-k\}}Q^{\deg C}.$$

Another advantage of the method in [29] is that it can be applied to every trellis section separately, which is convenient for time-varying codes. The decoding complexity of a particular code can also be decreased using methods in [30]. The complexity of the BCJR decoding algorithm has the same order as in (39) as well.

Symbol-wise decoding of a skew convolutional code C can be implemented using a trellis of the dual code $C^{⊥}$, see [31,32,33]. The order of decoding complexity in this case is also given in (39).

## 7. Conclusions

A new class of non-binary skew convolutional codes was defined that extends the class of fixed convolutional codes. The skew convolutional codes are equivalent to periodic time-varying classical convolutional codes but have as compact a description as fixed convolutional codes.

Given a field $F=F_{p^{M}}=F_{q^{m}}$ of characteristic *p* and code parameters n,k and μ; for every authomorphism $\theta \left(a\right)=q^{a}$ of the field, the subclass SCC(θ) of skew convolutional [n,k] codes of memory μ over the field is defined. All the subclasses have the same number of codes. In case of the identity automorphism θ=id, we obtain the subclass SCC(id) of classical fixed convolutional codes. Any other automorphism θ of the field gives a subclass SCC(θ) of skew convolutional codes that can be represented as a periodic time-varying convolutional code with typical period *m*. The total number of the subclasses SCC(θ) is equal to the number of divisors of *M*, which is usually not a large number. The class of *m*-periodic time-varying convolutional codes is larger than the class of skew convolutional codes. Every code in the subclass SCC(θ) is defined by a k×n polynomial generator matrix G(D) over the ring of θ-skew polynomials; hence, the descriptions of skew codes and fixed codes are the same, and the description is given by the same matrix G(D).

Every τ-periodic convolutional [n,k] code can be written as a fixed [τn,τk] code; hence, skew convolutional codes can be analyzed by methods known for fixed codes. We showed how to design generator and parity check matrices in polynomial and scalar forms, encoders and code trellises for skew convolutional codes, and their duals. Using code trellises for original and dual codes, in the case of channels without memory, one can apply Viterbi or BCJR decoding algorithms, or the dualized BCJR algorithm.

*Future work*. We gave just a first encounter with skew convolutional codes. There are many open problems remaining. The algebraic structure of classical fixed convolutional codes is well understood, see, e.g., [20,21] and references therein. The questions such as how to obtain a canonical generator matrix of a skew convolutional code and its dual, or how to design encoders of a fractional generator matrix can be considered in the future. Another open problem is to find good skew convolutional codes reaching an upper bound on the free distance. One possibility to obtain skew convolutional codes is based on unwrapping skew quasi-cyclic (QC) block codes (see such codes in [17]) in a way similar to [34] or [35], where it is shown how fixed classical convolutional codes can be obtained by unwrapping QC block codes and vice versa.
